# Therapeutic Outcome of Second Primary Malignancies in Patients with Well-Differentiated Thyroid Cancer

**DOI:** 10.1155/2016/9570171

**Published:** 2016-03-29

**Authors:** Miaw-Jene Liou, Ngan-Ming Tsang, Chuen Hsueh, Tzu-Chieh Chao, Jen-Der Lin

**Affiliations:** ^1^Division of Endocrinology and Metabolism, Department of Internal Medicine, Chang Gung Memorial Hospital, Chang Gung University, No. 5, Fu-Shin Street, Guishan District, Taoyuan 333, Taiwan; ^2^Department of Radiation Oncology, Chang Gung Memorial Hospital, Chang Gung University, No. 5, Fu-Shin Street, Guishan District, Taoyuan 333, Taiwan; ^3^Department of Pathology, Chang Gung Memorial Hospital, Chang Gung University, No. 5, Fu-Shin Street, Guishan District, Taoyuan 333, Taiwan; ^4^Department of General Surgery, Chang Gung Memorial Hospital, Chang Gung University, No. 5, Fu-Shin Street, Guishan District, Taoyuan 333, Taiwan

## Abstract

*Background.* The aims of this study were to analyze the clinical characteristics of SPM in patients with well-differentiated thyroid cancer and to determine the long-term prognosis in patients with double malignancies.* Materials and Methods.* We retrospectively analyzed 2,864 patients with well-differentiated thyroid cancer and a mean age of 44.0 ± 14.4 years. Of these, 200 (7.0%) were diagnosed with SPM, 115 of which were diagnosed with metachronous SPM.* Results.* Of 2,864 patients, 163 (5.7%) patients died of thyroid cancer and 301 (10.5%) died of any cause by the end of the follow-up period. Multivariate analysis identified age, SPM, external radiotherapy, TNM stage, and postoperative serum Tg level to be factors independently associated with decreased survival. Of 200 patients with SPM, 74 (37.0%) died. In comparison to the anachronous and synchronous groups, the metachronous SPM group had a higher mean age; more advanced tumor, node, and metastasis stage; lower remission rate; higher postoperative radioactive iodide (^131^I) accumulated dose; a higher proportion of patients who underwent external radiotherapy; and higher thyroid cancer and total mortality rates.* Conclusions.* Patients with well-differentiated thyroid carcinoma and metachronous SPM had worse prognoses compared to patients without SPM.

## 1. Introduction

Thyroid cancer is the most common endocrine cancer and one of the malignancies with the most rapidly increasing frequency over the last few decades [1–3]. Majority of thyroid cancers are well-differentiated and include the papillary and follicular types [4]. Most patients with well-differentiated thyroid carcinoma have excellent prognoses after appropriate therapy. To avoid the over- or underadministration of postthyroidectomy therapeutic modalities in patients with thyroid cancer, the stratification of the risk of recurrence and thyroid cancer mortality is urgently needed [5, 6]. An elevated risk of second primary malignancy (SPM), possibly related to radioactive iodide (^131^I), was recently reported in patients with thyroid cancer [7, 8]. During the follow-up period, the diagnosis and identification of SPM risk factors in patients with well-differentiated thyroid cancer are important. The development of SPM may influence the prognosis in patients with thyroid cancer. The purpose of this study was to analyze the clinical characteristics of SPM in patients with well-differentiated thyroid cancer of follicular cell origin and to determine the long-term prognosis in patients with double malignancies.

## 2. Materials and Methods

Between 1984 and 2013, 2,864 patients with well-differentiated thyroid carcinoma including 2,568 with papillary, 254 with follicular, and 42 with Hürthle cell carcinoma underwent thyroidectomy and long-term follow-up at Chang Gung Memorial Hospital in Linkou, Taiwan. The thyroid carcinomas were pathologically classified according to the 2004 World Health Organization criteria [9]. After the operation, all patients were staged using the American Joint Committee on Cancer-Tumor-Node-Metastasis (TNM) criteria (7th edition) [10]. In our center, most patients who had well-differentiated thyroid carcinoma with a tumor size > 1 cm underwent total thyroidectomy. Central or lateral dissection was performed for clinically enlarged lymph nodes or an extrathyroidal extension.

Thyroid remnant ablation was performed 4–6 weeks after thyroidectomy in 1,168 of 2,864 patients with well-differentiated thyroid carcinoma. The ^131^I ablation dose for most high-risk patients was 3.7 GBq (100 mCi). A whole-body scan (WBS) was performed 1 week after ^131^I administration. Levothyroxine (LT4) treatment was then initiated to reduce the thyroid-stimulating hormone (TSH) level without inducing clinical thyrotoxicosis. Therapeutic doses were in the range of 3.7–7.4 GBq (100–200 mCi) for cases of locoregional recurrence or distant metastasis. According to the radiation regulations in Taiwan, patients receiving < 1.1 GBq are classified as outpatients.

In patients who did not have a detectable ^131^I uptake beyond the thyroid bed during postablation WBS, thyroid hormone treatment was withdrawn after 6–12 months, and thyroglobulin (Tg), TSH, and anti-Tg antibody measurements were performed. SPM was diagnosed in patients with thyroid cancer based on the malignancy diagnosis code, defined by codes from 140 to 208.91 in ICD-9 clinical modification format. Patients with malignant neoplasms required diagnostic validation by at least two specialists based on medical record examination, laboratory and imaging results, and histological or cytological analyses. Patients diagnosed with malignant neoplasms were categorized into different groups according to the anatomic organ system. Depending on the timing of diagnosis, SPM was categorized into two groups: (1) anachronous (diagnosed 6 months before thyroid cancer diagnosis) or synchronous and (2) metachronous (diagnosed 6 months after thyroid cancer diagnosis). All subjects were Chinese residents of Taiwan. The study was approved by the Institutional Review Board of Chang Gung Memorial Hospital (CGMH).

The thyroid cancer database of CGMH in Linkou was established in 1995 and updated every 1-2 years. Data on patient age, sex, primary tumor size, ultrasonography results, fine needle aspiration cytology results, and thyroid function before and after surgery; surgical methods, histopathological findings, and TNM staging; 4–6 weeks' postoperative serum Tg levels, anti-Tg antibody levels, diagnostic results, therapeutic ^131^I scans, ^131^I accumulated dose, and chest radiography findings; and clinical status for the analysis of distant metastases via noninvasive radiological and nuclear medical studies, external radiotherapy site and dose, treatment outcomes, SPM diagnosis date and histopathology, causes of death, and survival status were recorded. At the end of 2013, patients were categorized into thyroid cancer remission and nonremission groups. Nonremission was defined as cytopathologically proven residual or recurrent status or positive imaging studies with detectable Tg after the withdrawal of thyroxin treatment.

Categorical data were compared using the Pearson chi-squared or Fisher's exact test for small size datasets. Continuous data were compared between the groups using unpaired *t*-test. Total and thyroid cancer-related mortalities were calculated, and the follow-up period was extended from the date of diagnosis to the date of last cancer-related mortality among patients who were followed up. Survival rates were calculated using the Kaplan-Meier method and compared using the log-rank test [11]. A multivariate Cox proportional hazards regression model was used to estimate the mortality risk. All statistical analyses were performed using SPSS version 17.0 statistical software (SPSS Inc., Chicago, IL, USA). *p* values < 0.05 were considered statistically significant in all tests.

## 3. Results

The 2,864 patients with well-differentiated thyroid carcinoma, 2,256 (78.0%) of which were women, had a mean age of 44.0 ± 14.4 years. The mean tumor size was 2.4 ± 1.7 cm, and 84.7% (2,425/2,864) of the patients underwent total thyroidectomy with or without lymph node dissection. A total of 1,950 (68.1%) cases were categorized as TNM stage I (Table 1). Over a mean follow-up period of 9.5 ± 6.7 years, 163 (5.7%) patients died of thyroid cancer. However, 301 (10.5%) patients had died of any cause by the end of the follow-up period.

Table 1 shows the clinical features of patients with well-differentiated thyroid cancer and those with and without SPM. The SPM group had a greater proportion of men, higher mean age, more advanced TNM stage, higher nonremission rate, higher ^131^I accumulated dose, higher proportion of patients who underwent external radiotherapy, and higher total and thyroid cancer mortality rates. Over a mean follow-up period of 9.2 ± 6.6 years, 74 (37.0%) patients died (Table 1).  Table 2 illustrates the multivariate analysis by Cox proportional hazards regression model for survival and overall mortality of the 2,864 patients with well-differentiated thyroid cancer. Age, SPM, external radiotherapy, TNM stage, and postoperative serum Tg level were significantly higher in the mortality group.

**Figure 1:**
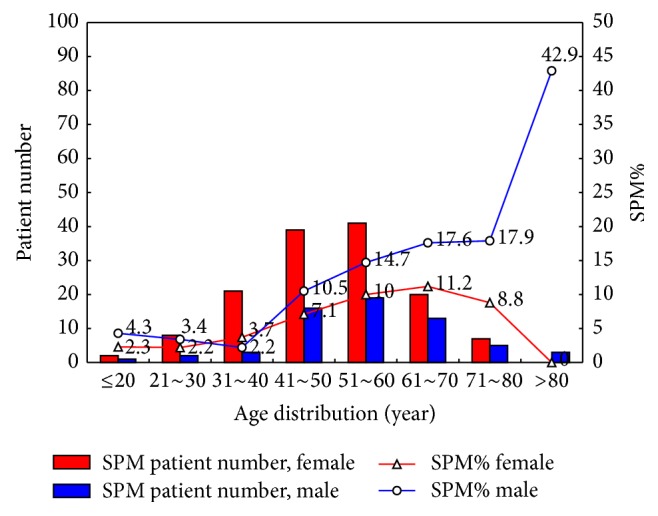
Number and percentage of second primary malignancies by age and sex.

**Figure 2:**
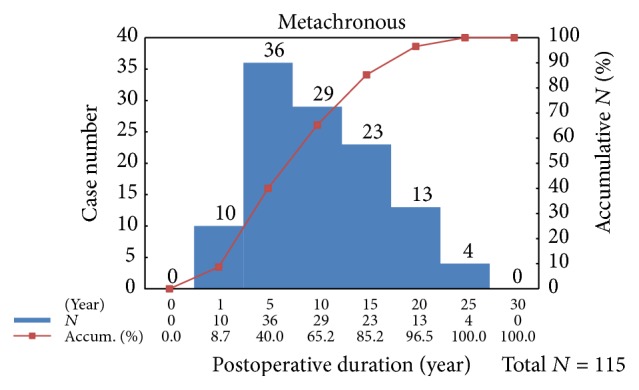
Case number of metachronous SPM after thyroidectomy.

Figure 1 illustrates the age and sex distribution of SPM cases by number and percentage. Two hundred patients were diagnosed with SPM. The incidences of thyroid cancer and SPM peaked at patient ages of 30–40 years and 50–60 years, respectively. Among the 200 patients with SPM, 115 (57.5%) were diagnosed with metachronous SPM. Forty percent of metachronous SPM cases were diagnosed in the 5 years after thyroid cancer treatment (Figure 2). The histological pattern and organ system of SPM are shown in Supplemental Table A, in Supplementary Material available online at http://dx.doi.org/10.1155/2016/9570171. Of the three leading histological patterns of SPM, the oral cavity and pharynx were more commonly affected in the anachronous or synchronous group. In contrast, the digestive system was more commonly affected in the metachronous group. The incidence of breast cancer was similar between the two groups. Supplemental Figure illustrated age and case number of top three metachronous SPMs. To discern the influence of thyroid cancer and treatment on the metachronous SPM group, we compared the clinical characteristics of patients with metachronous SPM to those with papillary thyroid cancer (Supplemental Table B). The metachronous SPM group had a higher mean age, less aggressive surgical treatment, more advanced TNM stage, higher nonremission rate, higher mean postoperative ^131^I accumulated dose, higher proportion of patients who underwent external radiotherapy, and higher thyroid cancer and total mortality rates. In addition, overall mortality was higher in patients with metachronous SPM compared to those with anachronous or synchronous SPM; however, the difference was not statistically significant (42.6% versus 29.4%, *p* = 0.056).

**Figure 3:**
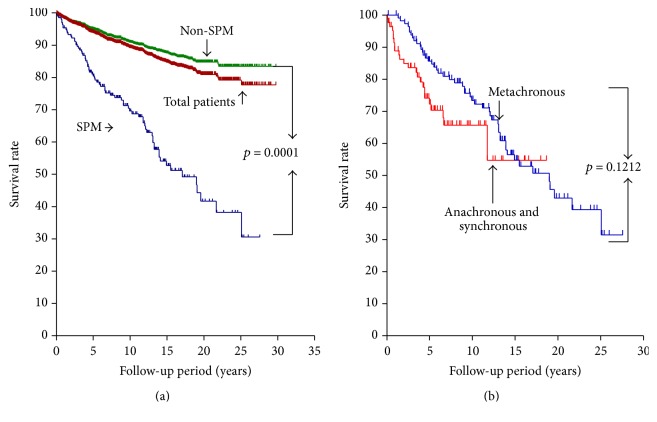
(a) Kaplan-Meier survival curves of thyroid cancer and overall mortality of the SPM and non-SPM patients. (b) Overall survival rates between metachronous group and anachronous with synchronous group.

Figure 3(a) illustrates Kaplan-Meier survival curves of thyroid cancer and overall mortality of the SPM and non-SPM patients. For all patients, those with SPM, and those without SPM, the 5-year survival rates were 93.5%, 80.4%, and 95.0%, respectively; the 10-year survival rates were 89.3%, 69.6%, and 90.9%, respectively; and the overall survival rates were 80.9%, 41.7%, and 84.8%, respectively. There was a statistically significant difference between the SPM and non-SPM groups (*p* = 0.0001). In contrast, there was no statistical difference of overall survival rates between metachronous group and anachronous with synchronous group (Figure 3(b)). Breast cancer was the most common SPM in patients with thyroid cancer, followed by the digestive system cancers. Among the 45 patients with breast cancer, 18 were diagnosed before or during thyroid cancer treatment. All the patients with thyroid cancer and breast cancer were female. Compared to the other SPM group, the breast cancer group had a lower mean age, lower nonremission rate, lower ^131^I accumulated dose, and lower proportion of patients who underwent external radiotherapy (Table 3). In addition, the thyroid cancer and total mortality rates were lower in breast cancer patients compared to those with other SPMs.

## 4. Discussion

SPM is reported to be involved in approximately 18% of cases of malignant tumor in the United States [12]. Most patients with well-differentiated thyroid cancer have good prognoses; however, SPM in patients with thyroid cancer may be a major cause of mortality and serious morbidity among thyroid cancer survivors. In this study, 4.01% (115/2,864) of patients with well-differentiated thyroid carcinoma also had metachronous SPM. This ratio is higher compared to that recently reported by a study conducted on a large patient cohort with thyroid cancer in Korea (1.6%; 2,895/178,844 patients with thyroid cancer) [7]. In addition, the SPM cancer type was different in these studies. In our study, the incidences of leukemia (4.3%) and lymphoma (3.5%) were low in patients with metachronous SPM. A higher incidence of SPM (13.04%) in patients with thyroid cancer was reported by Zafon et al. [13]. The results of their study are similar to ours in that male patients and older patients were more likely to have SPM. The reason for this ethnic and geographic diversity in SPM in patients with thyroid cancer requires further investigation.

Our data illustrated that SPM in patients with well-differentiated thyroid carcinoma presented with a more advanced TNM stage and higher thyroid cancer-specific and overall mortality compared to patients without SPM. In a recent study, breast cancer was the most common synchronous or antecedent nonthyroidal malignancy in women with well-differentiated thyroid carcinoma [14, 15]. SPM of digestive system, oral cavity, or pharynx origin was more common in this area than in Western countries [16].

As indicated in our study, incidental thyroid cancer (ITC) may be diagnosed, followed by anachronous or synchronous SPM of the oral cavity and pharynx. ITC may present during neck surgery for nonthyroid disease [17]. In a recent study, of the 690 patients with head and neck cancer, 234 (33.9%) had incidental thyroid lesions on ultrasonography, 9.4% of which were definitively diagnosed as thyroid cancer. Occasional papillary thyroid carcinoma occurred in the lymph nodes of patients with head and neck cancer who underwent radical neck dissection. Characteristic findings of thyroid origin demonstrated thyroid follicular structure and positive Tg staining on immunohistochemical analysis [18].

Controversy concerning the effect of ^131^I treatment on the occurrence of SPM in patients with thyroid cancer persists [8, 19, 20]. Previous studies had different study designs, varying follow-up periods, and different ^131^I dosages, which made an accurate comparison difficult. A meta-analysis of two multicenter studies by Blumhardt et al. concluded that the risk of SPM in thyroid cancer patients treated with ^131^I is slightly higher compared to the risk in thyroid cancer survivors not treated with ^131^I [21]. Our study showed that patients with metachronous SPM and thyroid cancer received higher accumulated doses compared to patients without SPM. In our study, after a mean follow-up period of 11.7 ± 6.6 for patients with metachronous SPM and thyroid cancer, the total mortality rate was 42.6%. We therefore agree with the recommendation from Tuttle's group that ^131^I therapy needs to be “rationed” in low-risk patients with well-differentiated thyroid cancer [20].

In our study, all the patients with breast cancer in the SPM group were female. Female sex is known as the dominant characteristic of patients with well-differentiated thyroid cancer. There were 2,256 (78.8%) female patients in our study group. Of the 139 women with SPM, 45 (32.4%) had breast cancer; 18 of them were diagnosed with it before or concomitantly with the thyroid cancer. A recent study showed that radiotherapy for breast cancer is significantly associated with an increased risk of a second nonbreast cancer both overall and in organs adjacent to the previous treatment fields [22]. Consistent with previous studies, in our study, synchronous primary cancers of the thyroid and breast were common findings [15]. A recent study showed that ^131^I therapy for thyroid cancer did not increase the incidence or recurrence rate of breast cancer [23]. Consistent with our data, the accumulated dose of ^131^I in patients with breast cancer and SPM was significantly lower than that in other patients with SPM. In comparison to a previous study, breast cancer was the most common synchronous and antecedent nonthyroidal malignancy in patients with thyroid cancer [14]. In contrast, prostate cancer, melanoma, and renal cell carcinoma are unusual SPMs in this area. SPMs of oral cavity, pharynx, or digestive system origin were more common. Breast, colon, and lung cancer are the three most prevalent types of cancer among women in Taiwan [24]. In our study, approximately 80% of patients were women. In addition, the histological type of the SPM in our patients was similar to that in the general population. This information might indicate that no specific cancer was the result of thyroid cancer treatment and the application of ^131^I and external radiation in our study did not increase specific SPM histological pattern.

## 5. Conclusion

SPM is common in patients with well-differentiated thyroid cancer. The overall mortality in patients with thyroid cancer and SPM may increase to 4.4 times higher than that of patients without SPM. Breast cancer and digestive system malignancies are the most common SPMs. During long-term follow-up, sites of high SPM incidence need to be more closely examined.

## Supplementary Material

Supplemental table A illustrated clinical features of 200 secondary primary malignancy (SPM) patients in anachronous with synchronous and metachronous two groups. Supplemental table B showed the difference between no SPM or anachronous with synchronous patients and metachronous SPM patients. Supplemental figure showed age distribution and case number of top three metachronous SPM.

## Figures and Tables

**Table 1:** Clinical features of 2,864 cases of well-differentiated^*∗*^ thyroid cancer with or without second primary malignancies (SPMs) between 1984 and 2013.

Clinical characteristic	Total	With SPM	Without SPM	*p*
Patient number	2,864	200 (7.0%)	2,664 (93.0%)	
Gender (female)	2,256 (78.8%)	139 (69.5%)	2,117 (79.5%)	0.0009
Age at diagnosis (year)	44.0 ± 14.4	51.6 ± 13.2	43.5 ± 14.4	0.0001
Mean tumor size (cm)	2.4 ± 1.7	2.4 ± 1.5	2.4 ± 1.7	0.6793
Thyroid operative method				0.0035
Total thyroidectomy	2425 (84.7%)	155 (77.5%)	2270 (85.2%)	
Less than total thyroidectomy	439 (15.3%)	45 (22.5%)	394 (14.8%)	
TNM stage				0.0001
Stage I	1950 (68.1%)	101 (50.5%)	1849 (69.4%)	
Stage II	287 (10.0%)	27 (13.5%)	260 (9.8%)	
Stage III	229 (8.0%)	25 (12.5%)	204 (7.6%)	
Stage IV	398 (13.9%)	47 (23.5%)	351 (13.2%)	
Nonremission	466 (16.3%)	48 (24.0%)	418 (15.7%)	0.0021
Follow-up period (year)	9.5 ± 6.7	9.2 ± 6.6	9.5 ± 6.7	0.5575
Postoperative ^131^I accumulative dose (mCi)	130.6 ± 193.6	166.3 ± 258.2	127.9 ± 187.6	0.0068
Radiation therapy	143 (5.0%)	24 (12.0%)	119 (4.5%)	0.0001
Multifocality	662 (23.1%)	50 (25.0%)	612 (23.0%)	0.5119
Mortality due to thyroid cancer	163 (5.7%)	18 (9.0%)	145 (5.4%)	0.0362
Overall mortality	301 (10.5%)	74 (37.0%)	227 (8.5%)	0.0001

^*∗*^Well-differentiated: including papillary, follicular, and Hürthle's cell thyroid cancer.

**Table 2:** Multivariate analysis by Cox proportional hazards regression model for survival and total mortality in 2,864 patients with well-differentiated thyroid cancer.

	*β* coefficient	Hazard ratio	95% confidence interval		*p* value
			Lower bound	Upper bound	
Age (year)	0.071	1.074	1.0598	1.0880	0.0000
Gender (F/M)	0.275	1.317	0.9743	1.7790	0.0733
SPM^*∗*^ (without/with SPM)	0.860	2.363	1.6987	3.2861	0.0000
Postop ^131^I accumulative dose (mCi)	0.000	0.999	0.9995	1.0004	0.7388
Radio therapy (no/yes)	1.289	3.628	2.5705	5.1209	0.0000
TNM stage (SI/SII/SIII/SIV)	0.260	1.305	1.1317	1.5056	0.0003
Postop 1-month Tg (ng/mL)	0.000	1.000	1.0000	1.0001	0.0000
Tumor size (cm)	0.0462	1.047	0.9896	1.1082	0.1099
Thyroid operative method (less than total/total thyroidectomy)	−0.2960	0.744	0.5023	1.1014	0.1395

^*∗*^SPM: second primary malignancy.

**Table 3:** Clinical features of 200 cases of well-differentiated thyroid cancer^*∗*^ with second primary malignancy (SPM) as breast cancer or other cancers.

Clinical characteristic	All patients	Breast cancer	Other cancers	*p* value
Patient number	200	45 (22.5%)	155 (77.5%)	
Gender (female)	139 (69.5%)	45 (100.0%)	94 (60.6%)	0.0001
Age at diagnosis (year)	51.6 ± 13.2	47.1 ± 10.2	52.9 ± 13.7	0.0099
Mean tumor size (cm)	2.4 ± 1.5	2.2 ± 1.2	2.4 ± 1.6	0.5271
Thyroid operative method				0.4470
Total thyroidectomy	155 (77.5%)	33 (73.3%)	122 (78.7%)	
Less than total thyroidectomy	45 (22.5%)	12 (26.7%)	33 (21.3%)	
TNM stage				0.0518
Stage I	101 (50.5%)	26 (57.8%)	75 (48.4%)	
Stage II	27 (13.5%)	9 (20.0%)	18 (11.6%)	
Stage III	25 (12.5%)	6 (13.3%)	19 (12.3%)	
Stage IV	47 (23.5%)	4 (8.9%)	43 (27.7%)	
Nonremission	48 (24.0%)	5 (11.1%)	43 (27.7%)	0.0215
Follow-up period (year)	9.2 ± 6.6	9.6 ± 6.0	9.1 ± 6.7	0.6467
Postoperative ^131^I accumulative dose (mCi)	166.3 ± 258.2	95.1 ± 102.1	187.0 ± 284.8	0.0357
^131^I dose ≥ 30 mCi	167 (83.5%)	40 (88.9%)	127 (81.9%)	0.2686
Radiation therapy	24 (12.0%)	1 (2.2%)	23 (14.8%)	0.0219
Time to metachronous SPM diagnosis (year) [range]	7.7 ± 5.7 [0.5–22.2]	7.5 ± 5.2 [0.6–21.2]	7.8 ± 5.8 [0.5–22.2]	0.8190
Multifocality	50 (25.0%)	10 (22.2%)	40 (25.8%)	0.6250
Mortality due to thyroid cancer	18 (9.0%)	1 (2.2%)	17 (11.0%)	0.0711
Overall mortality	74 (37.0%)	6 (13.3%)	68 (43.9%)	0.0002

^*∗*^Papillary, follicular, and Hürthle's cell thyroid cancer.
