# Supplementary material for: Therapeutic Outcome of Second Primary Malignancies in Patients with Well-Differentiated Thyroid Cancer
Source: Int J Endocrinol. 2016 Mar 29;2016:9570171. doi: 10.1155/2016/9570171 (PMC4828550; doi:10.1155/2016/9570171)
Supplement: Supplementary file 1 — Supplemental table A illustrated clinical features of 200 secondary primary malignancy (SPM) patients in anachronous with synchronous and metachronous two groups. Supplemental table B showed the difference between no SPM or anachronous with synchronous patients and metachronous SPM patients. Supplemental figure showed age distribution and case number of top three metachronous SPM. [file 9570171.f1.pdf]

SUPPLEMENTAL TABLE A: Second primary malignancy depending on timing of diagnosis in 200 patients with well-differentiated thyroid cancer

Neoplasm	All Patients	Anachronous and Synchronous	Metachronous
**All sites**	200	85 (42.5%) [71 ; 14]	115 (57.5%)
**Oral cavity and pharynx**	28 (14.0%)	22 (25.9%) [17 ; 5]	6 (5.2%)
**Digestive system**	44 (22.0%)	14 (16.5%) [14 ; 0]	30 (26.1%)
**Respiratory system**	18 (9.0%)	2 (2.4%) [0 ; 2]	16 (13.9%)
**Bones and joints**	2 (1.0%)	0	2 (1.7%)
**Soft tissue**	3 (1.5%)	1 (1.2%) [0 ; 1]	2 (1.7%)
**Skin**	6 (3.0%)	3 (3.5%) [2 ; 1]	3 (2.6%)
**Breast**	45 (22.5%)	18 (21.2%) [16 ; 2]	27 (23.5%)
**Female genital system**	19 (9.5%)	10 (11.8%) [7 ; 3]	9 (7.8%)
**Male genital system**	5 (2.5%)	3 (3.5%) [3 ; 0]	2 (1.7%)
**Urinary system**	13 (6.5%)	8 (9.4%) [8 ; 0]	5 (4.3%)
**Endocrine system**	1 (0.5%)	0	1 (0.9%)
**Lymphoma**	6 (3.0%)	2 (2.4%) [2 ; 0]	4 (3.5%)
**Leukemia**	6 (3.0%)	1 (1.2%) [1 ; 0]	5 (4.3%)
**Miscellaneous**	4 (2.0%)	1 (1.2%) [1 ; 0]	3 (2.6%)

Anachronous, diagnosed 6 months before thyroid cancer diagnosis; synchronous, and metachronous, diagnosed 6 months after thyroid cancer diagnosis

SUPPLEMENTAL TABLE B: Clinical features of 2,864 cases of well-differentiated thyroid cancer classified into metachronous and non-metachronous groups

Clinical characteristic	No SPM or anachronous/synchronous SPM	Metachronous SPM	*P*
**Patient number**	2,749 (96.0%)	115 (4.0%)	
**Gender (female)**	2172 (79.0%)	84 (73.0%)	0.1252
**Age at diagnosis (year)**	43.7 ± 14.4	50.6 ± 12.5	0.0001
**Mean tumor size (cm)**	2.4 ± 1.7	2.5 ± 1.5	0.7649
**Thyroid operative method**			0.0270
**Total thyroidectomy**	2336 (85.0%)	89 (77.4%)	
**Less than total thyroidectomy**	413 (15.0%)	26 (22.6%)	
**TNM stage**			0.0044
**Stage I**	1889 (68.7%)	61 (53.0%)	
**Stage II**	272 (9.9%)	15 (13.0%)	
**Stage III**	214 (7.8%)	15 (13.0%)	
**Stage IV**	374 (13.6%)	24 (20.9%)	
**Non-remission**	434 (15.8%)	32 (27.8%)	0.0006
**Follow-up period (year)**	9.4 ± 6.7	11.7 ± 6.6	0.0002
**Post-operative ^{131}I accumulative dose (mCi)**	127.3 ± 187.0	209.8 ± 302.0	0.0001
**^{131}I dose >= 30 mCi**	2376 (86.4%)	101 (87.8%)	0.6682
**Radiation therapy**	129 (4.7%)	14 (12.2%)	0.0003
**Multifocality**	635 (23.1%)	27 (23.5%)	0.9248
**Mortality due to thyroid cancer**	150 (5.5%)	13 (11.3%)	0.0080
Overall **mortality**	252 (9.2%)	49 (42.6%)	0.0001

Anachronous, diagnosed 6 months before thyroid cancer diagnosis; synchronous, and metachronous, diagnosed 6 months after thyroid cancer diagnosis

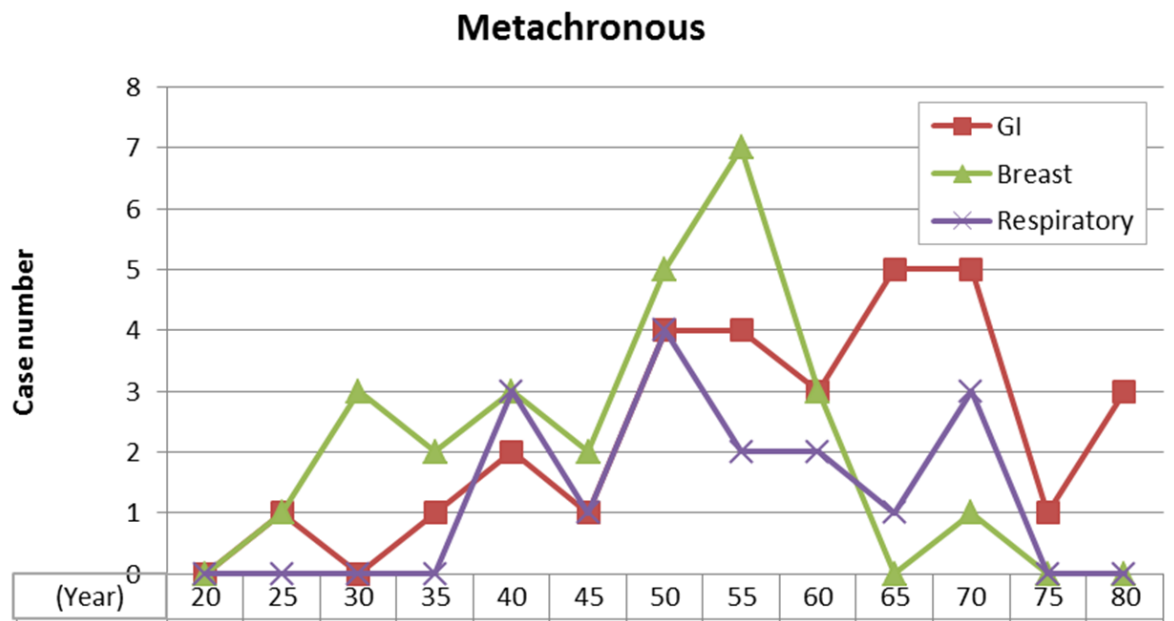

(Year)	20	25	30	35	40	45	50	55	60	65	70	75	80
GI	0	1	0	1	2	1	4	4	3	5	5	1	3
Breast	0	1	3	2	3	2	5	7	3	0	1	0	0
Respiratory	0	0	0	0	3	1	4	2	2	1	3	0	0

Age at diagnosis (year)

Total N= 73: GI cancer, N=30; Breast cancer, N=27; Respiratory system cancer, N=16

SUPPLEMENTAL FIGURE: Age distribution and case number of top three metachronous secondary primary malignancy.
